# MicroRNA Expression Analysis in the Cellulosic Biofuel Crop Switchgrass (*Panicum virgatum*) under Abiotic Stress

**DOI:** 10.1371/journal.pone.0032017

**Published:** 2012-03-28

**Authors:** Guiling Sun, C. Neal Stewart, Peng Xiao, Baohong Zhang

**Affiliations:** 1 Department of Biology, East Carolina University, Greenville, North Carolina, United States of America; 2 Department of Plant Sciences, University of Tennessee, Knoxville, Tennessee, United States of America; 3 BioEnergy Science Center, Oak Ridge National Laboratory, Oak Ridge, Tennessee, United States of America; 4 Department of Mathematics, East Carolina University, Greenville, North Carolina, United States of America; National Taiwan University, Taiwan

## Abstract

Switchgrass has increasingly been recognized as a dedicated biofuel crop for its broad adaptation to marginal lands and high biomass. However, little is known about the basic biology and the regulatory mechanisms of gene expression in switchgrass, particularly under stress conditions. In this study, we investigated the effect of salt and drought stress on switchgrass germination, growth and the expression of small regulatory RNAs. The results indicate that salt stress had a gradual but significant negative effect on switchgrass growth and development. The germination rate was significantly decreased from 82% for control to 36% under 1% NaCl treatment. However, drought stress had little effect on the germination rate but had a significant effect on the growth of switchgrass under the severest salinity stress. Both salt and drought stresses altered the expression pattern of miRNAs in a dose-dependent manner. However, each miRNA responded to drought stress in a different pattern. Salt and drought stress changed the expression level of miRNAs mainly from 0.9-fold up-regulation to 0.7-fold down-regulation. miRNAs were less sensitive to drought treatment than salinity treatment, as evidenced by the narrow fold change in expression levels. Although the range of change in expression level of miRNAs was similar under salt and drought stress, no miRNAs displayed significant change in expression level under all tested salt conditions. Two miRNAs, miR156 and miR162, showed significantly change in expression level under high drought stress. This suggests that miR156 and miR162 may attribute to the adaption of switchgrass to drought stress and are good candidates for improving switchgrass as a biofuel crop by transgenic technology.

## Introduction

Switchgrass (*Panicum virgatum* L.) is a warm-season perennial grass that is native to North America. It has been widely used as a forage crop and thrives well on marginal lands and can tolerate semi-arid conditions. Its broad adaptation and rapid growth rate provide stable and high supply of biomass for biofuel production [1], [2]. Despite of its increasing importance as a biofuel crop, we still know very little about the basic biology of switchgrass under abiotic stress conditions, such as those posed by salt and drought; important characteristics to characterize include seed germination, plant growth, and the regulation mechanism of gene expression when plants are under stress. Such baseline data are needed to gauge the effects of genetic improvements and to guide researchers to appropriate gene candidates to manipulate for improving stress tolerance.

MicroRNAs (miRNAs) are an extensive class of newly discovered non-coding small RNAs that regulate gene expression at the post-transcription levels by mRNA cleavage or translation repression [3], [4]. By regulating their target proteins, miRNAs have been reported to be involved in diverse biological processes, including organ development [5], [6], hormone signaling [7], defense against pathogens [8], and response to abiotic and biotic stresses [9], [10], [11]. Important abiotic stresses in this regard include salinity [12], drought [13], [14], cold [15], and heavy metals [16], nutrition, and other stresses [11].

More than 40 miRNA families have been associated with abiotic stress in plants [17], 13 of which have been found to be responsive to salt and drought stresses [14]. These 13 miRNAs include miR156, miR159, miR165, miR167, miR168, miR169, miR319, miR393, miR395, miR396, miR398, miR399, and miR402 [14]. Recently, miR172 and miR397 were also reported to be implicated in drought stress in *Solanum* and rice [18], [19]. Almost all of these stress-induced miRNAs are evolutionarily conserved, which suggests that miRNAs-mediated regulatory mechanism may be evolutionarily conserved for corresponding environmental stresses in plants. However, the same miRNAs reported to respond abiotic stress in one certain species may not have the same function in other species. To date, opposite expression in Arabidopsis and rice under drought stress has been observed for at least 10 miRNAs that involve in stress response [19], [20], [21]. This raises the question whether these reported stress responsive miRNAs still play tolerance roles in other plant species.

miRNAs in switchgrass have been recently studied using computational and experimental approaches [22], [23]. Although the expression level of up to 16 miRNAs were studied in seedling and adult development stages and in two different leave tissues by RNA blot experiment, no investigations have been performed on the expression patterns of miRNAs and their potential roles under stress conditions in this important biofuel feedstock. In this study, we investigated how salt and drought stresses affected the germination and biomass production of switchgrass and how these stresses altered the expression levels of miRNAs. We chose 12 miRNAs to study and these 12 miRNAs are conserved in dicots and monocots. Except miR162, 11 of the 12 miRNAs have been reported to be involved in salt or drought stress in previous studies in model plant species [17]. miR162 was also selected because of its important role in miRNA processing by negatively regulating the dicer-like 1 (DCL1) gene [24].

## Materials and Methods

### Plants

Switchgrass cv Alamo was used in this experiment. Alamo is a lowland cultivar of switchgrass adapted well to the southeastern United States [25], [26]. Seed surface sterilization was performed with 70% (v/v) ethanol for 60 s, 6% (v/v) bleach for 6–8 min, followed washed by sterile water 4 times. Healthy-appearing seeds were germinated on 1/2 Murashige and Skoog (MS) medium (pH 5.8) containing 0.8% agar under a 16 h light/8 h dark cycle at room temperature for 10 d. The media were supplemented with 0.1%, 0.25%, 0.5% and 1.0% NaCl to simulate increasing degrees of salinity stress and with 1%, 2.5%, 5% and 7.5% PEG to simulate varying degrees of drought stress. Each treatment was replicated for 5 time as in five individual plates and each plate contained 20 seeds. Germinated seeds were counted for all the plates; the root length and the weight of aboveground and underground parts were measured for each seedling. The aboveground parts were immediately frozen in liquid nitrogen after measurement.

### RNA isolation and real-time RT-PCR analysis

To avoid the individual difference in gene expression, at least four seedlings were used for total RNA extraction. Total RNA was isolated from the grinded samples using the mirVana™ miRNA Isolation Kit (Ambion, Austin, TX, USA) following the user manual. The quantity and quality of total RNA were assessed using a Nanodrop ND-1000 (Nanodrop Technologies, Wilmington, DE, USA) and gel electrophoresis.

Twelve miRNAs were selected for this study, which included miR156, miR 157, miR 159, miR 162, miR167, miR169, miR172, miR395, miR396, miR397, miR398, and miR399. A majority of these miRNAs have been reported to play a role under stress conditions in model plant species. Nine of these–miR156, miR167, miR169, miR172, miR395, miR396, miR397, miR398, and miR399–were identified in previous studies [22], [23]; the other three miRNAs were identified in the deep sequencing small RNA dataset. Table S1 listed the primers for these 12 miRNAs.

Real-time RT-PCR was used to characterize the expression of 12 miRNAs (for sequences, see Table S1) in aboveground biomass under salt and drought stress. Firstly, the reverse transcription (RT) reaction was carried out using the TaqMan® microRNA reverse transcription kit (Applied Biosystems, Foster City, CA, USA). The mixture of 12 miRNA specific RT primers (Table S1) was used to obtain the cDNAs of miRNAs. Second, real-time RT-PCR was run on an Applied Biosystems 7300 Sequence Detection System (Foster City, CA, USA) according to the manufacturer's protocol using gene specific primers. The gene specific primers of miRNAs were designed by following the manual of microRNA reverse transcription kit; their sequences are shown in Table S1.

Because there is no reliable reference gene for switchgrass miRNA analysis, to better present the results, the expression levels of each miRNA were calculated using the mean C_T_ values of the 12 studied miRNAs. Changes in expression level of miRNAs were assessed using the mean value of ΔCt. Each miRNA was conducted with three biological replicates for different stress treatments. Analysis of variance (ANOVA) of single factor was performed to compare the expression difference of miRNAs under different salt and drought treatments.

## Results

### The effect of salt and drought stresses on switchgrass growth and development

We studied several response variables in salt and drought stress treatments: switchgrass germination rate, biomass accumulation, development of leaves and roots, and gene expression dynamics of multiple miRNA genes. Salt stress significantly impacted switchgrass growth (Table 1). Under normal condition, the germination rate was 82±5.7%; however, as salinity concentrations increased (with the exception of the lowest concentration of 0.1%), the germination rate was significantly decreased (p<0.001) from 82±5.7% to 62–68% under moderate salinity (0.25% and 0.5%), and then to 36±8.2% at high salinity (1%). In addition to decreasing the germination rate, whole plant weight, aboveground- and belowground biomass, shoot∶root biomass ratio, and the root length of switchgrass seedlings also significantly decreased with increasing salinity in a dose-dependent manner, particularly at the high salinity treatment (Table 1). At low concentrations (0.1% and 0.25%), salinity stress did not greatly affect switchgrass growth and development, and the lowest (0.1%) salinity treatment even stimulated switchgrass growth with about 10% increase of their root length and biomass; 0.25% NaCl did not significantly affect switchgrass growth; however, high concentrations (0.5% and 1%) of NaCl, and particularly 1%, significantly inhibited switchgrass growth and development (p<0.001). At the 1% NaCl treatment, the switchgrass growth was inhibited about 50% on both aboveground and underground biomass; root elongation was also inhibited in the 1% NaCl treatment. Consistent with the effect of 1% salt concentration on root length, shoot length was also significantly reduced compared with those plants grown on the control and other low salinity stress (data not shown).The shoot∶root weight ratio initially decreased and then increased with increasing NaCl concentrations, suggesting that low NaCl concentrations (0.1 and 0.25%) inhibited more aboveground growth than root growth and high NaCl concentrations (0.5 and 1.0%) played a role in especially inhibiting root growth.

**Table 1:** Effect of salt and drought stress on the germination and growth of switchgrass.

Treatment		Germination rate	Weight of each seeding (mg)	Seedling aboveground biomass (mg)	Seedling belowground biomass (mg)	Abovegound to belowground biomass ration	Root length (cm)
NaCl	Control	82±5.7ab	6.9±0.92ab	5.3±0.44a	1.6±0.53ab	3.7±1.23ab	1.2±0.84a
	0.1%	83±7.58a	7.5±0.81a	5.4±0.41a	2.1±0.47a	2.7±0.63a	1.3±1.02a
	0.25%	68±5.7bc	6.5±0.72ab	4.7±0.43ab	1.8±0.43ab	2.7±0.55a	1.2±0.77a
	0.5%	62±10.37c	5.1±1.21bc	4±0.77bc	1.1±0.45ab	4.1±1ab	0.9±0.59ab
	1%	36±8.22d	3.9±1.33c	3±0.6c	0.9±1.07b	6.4±3.68b	0.6±0.5b
PEG	Control	83±4.47a	7.3±0.91a	5.5±0.69a	1.7±0.38a	3.3±0.61a	1.2±0.84ab
	1%	78±11.51a	6.6±1.08a	5.3±0.77ab	1.3±0.38a	4.3±0.79a	1.3±0.9a
	2.5%	76.3±8.54a	6.7±0.88a	5.3±0.61a	1.4±0.34a	3.9±0.7a	1.3±0.9a
	5%	68.8±14.36a	6±0.54a	4.9±0.45a	1.1±0.25ab	4.4±0.88a	1.1±0.67ab
	7.5%	77.5±6.45a	3.8±0.75b	3.3±0.41b	0.5±0.42b	9.4±4.6b	0.7±0.58b

Each treatment has five replicates. The result was shown in mean value and stand deviation. The data with the same letter shows no significant difference by ANOVA.

Drought stress did not have a great effect on switchgrass seed germination; however, it did significantly decrease switchgrass biomass (p<0.001, Table 1). Although the biomass accumulation and development of leaves and roots decreased gradually with the increase of PEG concentrations, only 5% and 7.5% PEG significantly inhibited switchgrass growth. The biomass accumulation and development of roots decreased by about 40–50% under 7.5% PEG concentration. However, the weight of underground biomass decreased about 70% under the highest PEG concentration compared with controls.

### Expression levels of miRNAs in switchgrass young seedlings

**Figure 1:**
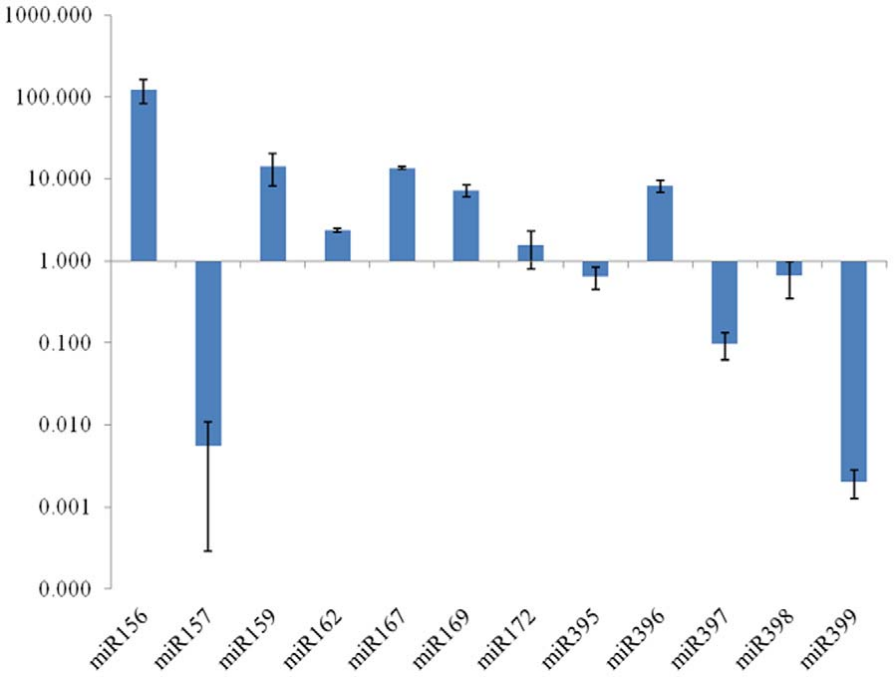
Relative expression levels of 12 miRNAs in 10 day-old switchgrass shoots. Fold change was normalized against the mean value of these 12 miRNAs. Error bars indicate standard error of three biological replicates.

All the 12 tested miRNAs were expressed in switchgrass young seedlings, but their expression level varied from each other (Figure 1). Among the assayed miRNAs, miR156 was the miRNA with the highest expression level; compared with other miRNAs, the expression level of miR156 was 124-fold of the average of the 12 miRNAs. miR159, miR167, miR169 and miR396 were also highly expressed in switchgrass young seedlings. However, the expression level of miR157, miR399 and miR397 were relatively low and their expression levels were less than 10% of the average expression level of the 12 tested miRNAs; of them, miR157 and miR399 were the miRNAs with the lowest expression. miR156 and miR157 were grouped into one miRNA family because of their high sequence similarity (Table S1) and shared targets. The significant difference of miR156 and miR157 in switchgrass indicates that they may be involved in different development stages and play different roles in switchgrass growth and development.

### Salt stress altered the expression pattern of miRNAs in switchgrass

**Figure 2:**
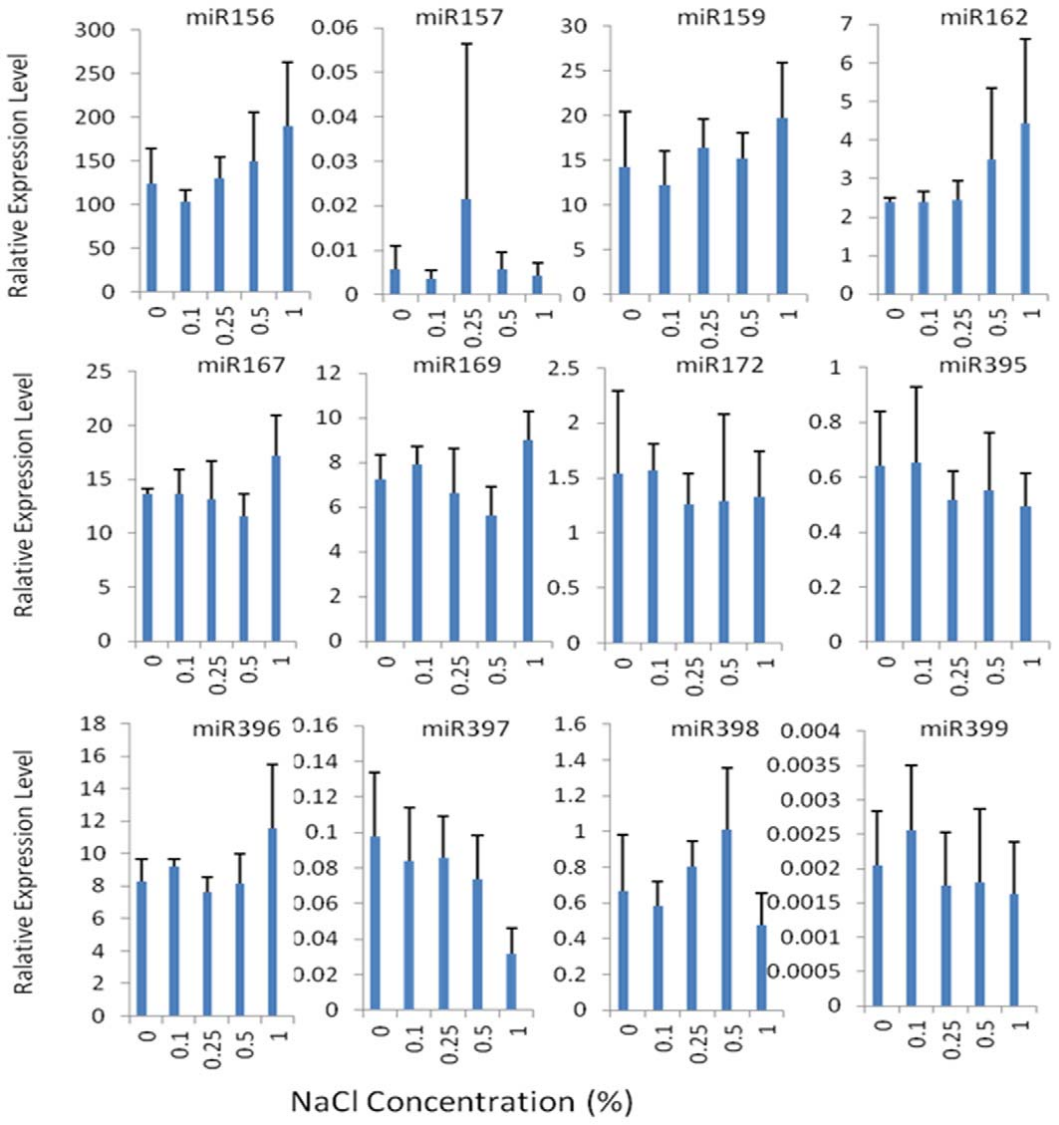
Expression analysis of 12 miRNAs in switchgrass shoots under NaCl treatment. Fold change was normalized against the mean value of these 12 miRNAs. Error bars indicate standard error of three biological replicates.

Salinity treatment affected the expression of miRNAs in switchgrass young seedlings with a dose-dependent manner (Figure 2). The expression of miR162 was increased as increasing salinity concentrations with the maximum 0.9-fold up-regulation under the highest tested salt stress condition (1%); in contrast, miR397 showed decrease in expression level with the increase of salt concentration with the maximum 0.7-fold down-regulation under the highest salt concentration. The expression level of miR156 and miR159 was down-regulated under 0.1% salt concentration, whereas it was up-regulated under higher salt concentration; in contrast, the expression level of miR172, miR395, and miR399 was up-regulated under 0.1% salt concentration while down-regulated under higher salt conditions. The expression level of miR157 and miR398 was down-regulated under lowest and highest salt concentration while up-regulated in the moderate salt stress. Of special interest, the expression level of miR167 was down-regulated under 0.5% or lower salt concentration while was up-regulated by 0.3-fold when exposed to 1% salt condition.

Although salinity treatment affected the expression of all tested miRNAs, the changes in miRNA expression were small. The highest fold change in expression was miR157 and only 2.8-fold up-regulation was observed under moderate salt treatment. All the other 11 miRNAs only showed less than 0.8-fold up-regulation in expression level and the 0.8-fold up-regulation was observed in miR162 under the most severe salt stress. miR397 was the most down-regulated of all miRNAs evaluated with a change in expression of 0.7-fold at 1% salt treatment; miR157 showed the second greatest change in expression of 0.4-fold down-regulated at 0.1% salt stress.

### Drought stress altered the expression pattern of miRNAs in switchgrass

**Figure 3:**
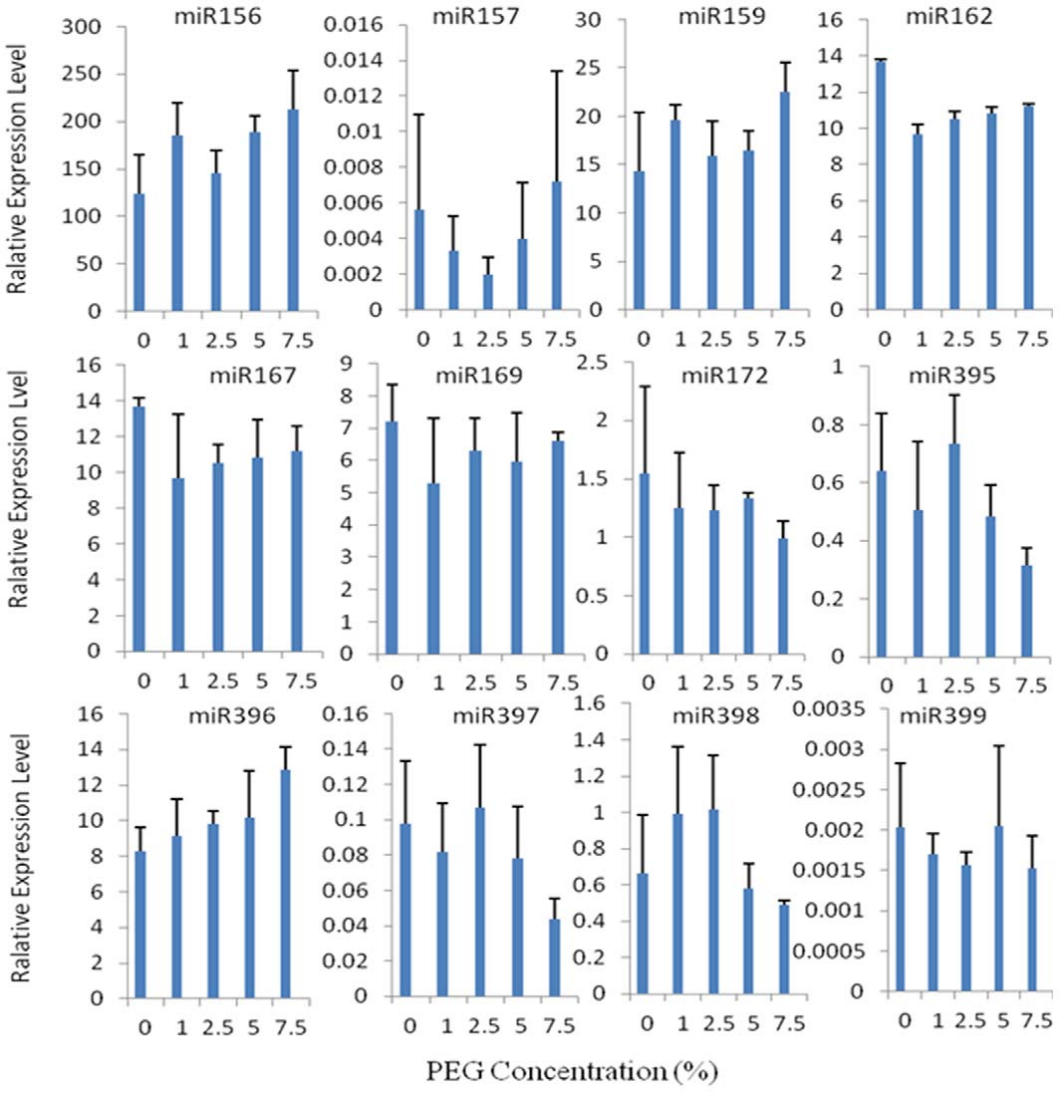
Expression analysis of 12 miRNAs in switchgrass shoots under PEG treatment. Fold change was normalized against the mean value of these 12 miRNAs. Error bars indicate standard error of three biological replicates.

Similar to those effects under salt stress, drought stress also altered the expression level of miRNAs in young switchgrass seedlings, and in a dose-dependent manner (Figure 3). miR156, miR159, and miR396 showed up-regulation expression levels under all drought conditions, with the greatest 0.9-fold change under 7.5% PEG; however, miR167, miR169, and miR172 displayed down-regulated expression level under all drought conditions, with the largest 1.5-fold down regulation under the most intensive drought condition. The expression level of miR157 was up-regulated by 0.3-fold under the highest 7.5% PEG but was down-regulated under other lower drought conditions with a maximum 0.6-fold change under 2.5% PEG concentration. The expression levels of miR395 and miR397 was only up-regulated under 2.5% PEG, with a maximum 0.5-fold change in miR398 under 2.5% PEG; in other drought condition, both miRNAs were down-regulated with a dose-dependent mannor, with the highest 0.5-fold change in miR395 under the 7.5% PEG concentration. For miR-398, it was up-regulated at lower concentrations (1% and 2.5%) and was down-regulated in high PEG treatment.

The expression of miRNAs was less sensitive to drought treatment than salinity treatment evidenced by the narrow fold change in expression levels. The expression change ranged from 0.9-fold up-regulation to 0.6-fold down-regulation after the drought treatments. The expression level of miR162 showed the highest 0.9-fold up-regulation under the highest drought stress; miR156 showed the second largest 0.7-fold change in expression level under 7.5% PEG; all the other 11 miRNAs shows less than 0.6-fold up-regulation in expression level. The largest down-regulated expression, 0.6-fold change, was observed in miR157 at 2.5% PEG and in miR397 at 7.5% PEG.

ANOVA analysis indicated that the expression level of two miRNAs, miR156 and miR162, significantly changed under high drought stress (p<0.001). Compared with the expression level under untreated control conditions, miR156 was significantly up-regulated under 7.5% PEG concentration; in contrast, the expression level of miR162 was significantly inhibited by 5% and 7.5% PEG treatment.

## Discussion

One reason why switchgrass has gained attention as a dedicated biofuel crop is that it can grow on suboptimal land that has relatively low available water. Our results indicated that salt stress had a significant effect on the germination rate and growth of switchgrass in almost all tested abiotic stresses. Interestingly, drought stress had no obvious effect on the germination rate of switchgrass; the significant effect of drought stress on switchgrass growth was observed only when switchgrass was exposed to high water stress conditions. Barney and colleagues also reported that switchgrass demonstrated great tolerance to drought stress [27]. This result suggests that switchgrass has evolved a more effective mechanism to cope with drought stress as opposed to salt stress. Therefore, it is interesting to further investigate the change in gene expression, especially the gene expression regulators, under such stress conditions.

miRNAs are an extensive class of newly discovered gene regulators. They have been reported to play important roles under abiotic stress in model plant species. Using qRT-PCR, we studied the expression change of 12 conserved miRNAs in 11 days old switchgrass seedlings exposed to salt and drought stress. Of the 12 miRNAs, 11 have been demonstrated to be involved in salt or drought stress in previous study, eight of these in both the dicot *Arabidopsis thaliana* and the monocot *Oryza sativa*. Our results indicate that both salt and drought stresses altered the expression pattern of miRNAs in a dose-dependent manner. Salt and drought stress changed the expression level of miRNAs mainly from 0.9-fold up-regulation to 0.7-fold down-regulation, and drought stress altered the expression of miRNAs from 0.9-fold up-regulation to 0.6-fold down-regulation. Although the range of change in expression level of miRNAs was similar under salt and drought stress, no miRNAs displayed significant change in expression level under all tested salt conditions, however, two miRNAs, miR156 and miR162, showed significantly change in expression level under high drought stress. This suggests that miR156 and miR162 may attribute to the adaption of switchgrass to drought stress and are good candidates for improving switchgrass as a biofuel crop by transgenic technology.

miR156 is one class of conserved miRNAs, which play an important role in multiple biological process. By targeting squamosal promoter binding protein-like (SPL) genes, miR156 has been demonstrated to temporally regulate shoot development [28], control the development timing from juvenile to adult transition together with miR172 [29], [30], secure male fertility [31], regulate anthocyanin biosynthesis [32], and is involved in flowering control [33]. Overexpression of miR156 in *Arabidopsis*, rice, and maize led to a prolonged vegetative phase together with the production of significantly higher number of total leaves, which resulted in enhanced biomass accumulation [34], [35], [36]. miR156 was demonstrated by microarray-based analysis to response to salt stress but not to drought stress in *Arabidopsis*; miR156 was induced by 1.6-fold by salinity stress [37]. In rice, miR156 was found to respond to drought stress and was down-regulated by 2.1-fold by drought stress [19]. However, study on salt stress of maize showed that miR156 was not involved in salt response [38]. Our results indicate that the expression of miR156 was significantly induced by 1.7 fold under high drought condition. Further studies on the expression change of downstream genes would help us to illustrate the mechanism of tolerance of switchgrass to drought stress.

miR162 has been reported to involve in miRNA biogenesis by negatively regulating dicer-like 1 (DCL1) gene [24]. It was also implicated to play a role in cotton fiber development [39]. In our study, miR162 is the only miRNA that has not been associated with salt or drought stress, although it was reported to be significantly down-regulated under cadmium stress in rice [40]. In our study, miR162 was down-regulated under all drought stress treatments, while the expression change was statistically significantly only under high drought conditions. This suggests that miR162 plays an important role during drought stress and feedback regulation of miRNAs also functions in switchgrass to adapt the drought stress. Given the multiple functions of miR156 and miR162, it would be interesting to investigate how the numerous phenotypes would play out in overexpressed transgenic switchgrass and whether these overexpressed miRNAs would confer higher tolerance to switchgrass.

## Supporting Information

Table S1 Primers used in reverse transcription (RT) and qPCR for amplifying 12 miRNAs. The reverse primer is provided by the kit. The nucleotides in green are the same as or complementary to the miRNA sequences. RT and FP in the primer name indicate that the primer is reverse transcription primer or forward PCR primer respectively.
(DOC)
