# Supplementary material for: MicroRNA Expression Analysis in the Cellulosic Biofuel Crop Switchgrass (Panicum virgatum) under Abiotic Stress
Source: PLoS One. 2012 Mar 28;7(3):e32017. doi: 10.1371/journal.pone.0032017 (PMC3314629; doi:10.1371/journal.pone.0032017)
Supplement: Table S1 — Primers used in reverse transcription (RT) and qPCR for amplifying 12 miRNAs. The reverse primer is provided by the kit. The nucleotides in green are the same as or complementary to the miRNA sequences. RT and FP in the primer name indicate that the primer is reverse transcription primer or forward PCR primer respectively. (DOC) [file pone.0032017.s001.doc]

Table S1. Primers used in reverse transcription (RT) and qPCR for amplifying 12 miRNAs. The reverse primer is provided by the kit. The nucleotides in green are the same as or complementary to the miRNA sequences. RT and FP in the primer name indicate that the primer is reverse transcription primer or forward PCR primer respectively.

| **miR** | **Sequence** | **Primer names** | | **Primer sequence** | | | |  |
| --- | --- | --- | --- | --- | --- | --- | --- | --- |
| miR 156 | UGACAGAAGAGAGUGAGCAC | miR156-7RT | GTCGTATCCAGTGCAGGGTCCGAGGTATTCGCACTGGATACGACGTGCTC | | | |  | |
|  |  | miR156FP | GCGGCGGTGACAGAAGAGAGT | | | |  | |
| miR 157 | UUGACAGAAGAUAGAGAGCAC | miR156-7RT | GTCGTATCCAGTGCAGGGTCCGAGGTATTCGCACTGGATACGACGTGCTC ACTGGATACGACGTGCTC | | | |  | |
|  |  | miR157FP | GCGGCGGTTGACAGAAGATAGA | | | |  | |
| miR 159 | UUUGGAUUGAAGGGAGCUCUA | miR159RT | GTCGTATCCAGTGCAGGGTCCGAGGTATTCGCACTGGATACGACTAGAGC | | | |  | |
|  |  | miR159FP | GCGGCGGTTTGGATTGAAGGG | | | |  | |
| miR 162 | UCGAUAAACCUCUGCAUCCAG | miR162RT | GTCGTATCCAGTGCAGGGTCCGAGGTATTCGCACTGGATACGACCTGGAT | | | |  | |
|  |  | miR162FP | GCGGCGGTCGATAAACCTCTG | | | |  | |
| miR 167 | UGAAGCUGCCAGCAUGAUCUA | miR167RT | GTCGTATCCAGTGCAGGGTCCGAGGTATTCGCACTGGATACGACTAGATC | | | |  | |
|  |  | miR167FP | GCGGCGGTGAAGCTGCCAGCA | | | |  | |
| miR 169 | CAGCCAAGGAUGACUUGCCGA | miR169RT | GTCGTATCCAGTGCAGGGTCCGAGGTATTCGCACTGGATACGACTCGGCA | | | |  | |
|  |  | miR169FP | GCGGCGGCAGCCAAGGATGAC | | | |  | |
| miR 172 | AGAAUCUUGAUGAUGCUGCAU | miR172RT | GTCGTATCCAGTGCAGGGTCCGAGGTATTCGCACTGGATACGACATGCAG | | | |  | |
|  |  | miR172FP | GCGGCGGAGAATCTTGATGAT | | | |  | |
| miR 395 | CUGAAGUGUUUGGGGGAACUC | miR395RT | GTCGTATCCAGTGCAGGGTCCGAGGTATTCGCACTGGATACGACGAGTTC | | | |  | |
|  |  | miR395FP | GCGGCGGCTGAAGTGTTTGGG | | | |  | |
| miR 396 | UUCCACAGCUUUCUUGAACUG | miR396RT | GTCGTATCCAGTGCAGGGTCCGAGGTATTCGCACTGGATACGACCAGTTC | | | |  | |
|  |  | miR396FP | GCGGCGGTTCCACAGCTTTCT | | | |  | |
| miR 397 | UCAUUGAGUGCAGCGUUGAUG | miR397RT | GTCGTATCCAGTGCAGGGTCCGAGGTATTCGCACTGGATACGACCATCAA | | | |  | |
|  |  | miR397FP | GCGGCGGTCATTGAGTGCAGC | | | |  | |
| miR 398 | UGUGUUCUCAGGUCACCCCUU | miR398RT | GTCGTATCCAGTGCAGGGTCCGAGGTATTCGCACTGGATACGACAAGGGG | | | |  | |
|  |  | miR398FP | GCGGCGGTGTGTTCTCAGGTC | | | |  | |
| miR 399 | UGCCAAAGGAGAUUUGCCCUG | miR399RT | GTCGTATCCAGTGCAGGGTCCGAGGTATTCGCACTGGATACGACCAGGGC | | | |  | |
|  |  | miR399FP | GCGGCGGTGCCAAAGGAGATT | | | |  | |
|  |  |  |  | | | |  | |
|  |  |  | | |  |  | | |
